# Preoperative Opioid Informed Consent and Prescribing Practices in Children Undergoing Orthopaedic Trauma Surgery

**DOI:** 10.5435/JAAOSGlobal-D-21-00309

**Published:** 2022-01-24

**Authors:** Brendan A. Williams, Lacey C. Magee, Christopher A. Makarewich, Ishaan Swarup, Lia W. McNeely, Apurva S. Shah

**Affiliations:** From the Department of Orthopaedics, The Children's Hospital of Philadelphia, Philadelphia, PA (Dr. Williams, Dr. McNeely, and Dr. Shah); the Department of Otolaryngology, Cleveland Clinic, Cleveland, OH (Dr. Magee); the Department of Orthopaedics, University of Utah, Salt Lake City, UT (Dr. Makarewich); and the Department of Orthopaedic Surgery, University of California San Francisco, San Francisco, CA (Dr. Swarup).

## Abstract

This study sought to examine prescribing practices for pediatric patients undergoing orthopaedic trauma surgery and assess the effect of state-mandated preoperative informed consent for opioids.

**Methods:**

A retrospective single-institution cohort study was done between 2016 and 2018 for surgically managed isolated orthopaedic trauma with cohorting based on the presence of preoperative opioid consent. Analyses examined cohort demographic and procedural factors associated with the number of opioid doses prescribed.

**Results:**

A total of 1,793 patients met the study criteria. The proportion of patients prescribed opioids (*P* = 0.0378) and the number of doses (*P* < 0.001) were lower in consented patients. Differences were greater among those receiving solution (versus tablets). No cohort differences were observed in refill needs. Nonopioid medications prescribing increased. Multivariate analysis identified multiple factors, including preoperative opioid consent (*P* = 0.013) associated with fewer prescribed opioid doses.

**Discussion:**

After the implementation of preoperative opioid consenting, patients were prescribed fewer opioid doses after pediatric orthopaedic trauma surgery. The increased utilization of nonopioid therapies was also evident. These changes occurred despite a shorter length of hospital stay and without changes in the studied proxies of postoperative pain control. An increased awareness of opioid risks through formal consent discussion may help to facilitate reduced reliance on opioids for children in the postoperative period.

Orthopaedic surgeons are a major contributor to opioid dispensing among pediatric patients, representing the fourth highest prescriber to adolescent patients in the United States.^1^ Early opioid exposure may pose long-term risks to pediatric patients, whereas leftover and unused medications may result in adolescent misuse.^2,3^ This, coupled with the frequent overprescription of these medications that has been identified in this population,^4,5^ places pediatric patients undergoing surgical treatment for orthopaedic trauma at considerable risk of opioid-related adverse events. Recent calls have been made by members of the Pediatric Orthopaedic Society of North America^6^ and the American Orthopaedic Association^7^ to standardize opioid prescribing practices and identify other strategies for perioperative pain management to reduce opioid consumption, but data to guide these efforts are lacking. Legislative actions have also been implemented to curb opioid overprescribing, such as Pennsylvania Act 125 mandating preoperative opioid consenting for minors,^8^ but their effects in the pediatric population have yet to be assessed.

The primary goal of this study was to evaluate opioid prescribing after orthopaedic trauma surgery at our institution and examine the relationship between preoperative opioid consenting and prescription patterns. We also sought to explore demographic, prescriber, and procedural factors associated with differences in opioid prescribing practices. We hypothesized that preoperative opioid consenting would be associated with a reduction in postoperative opioid prescribing. Findings from this study will evaluate opioid prescribing trends at a single center in addition to evaluating the effect of informed consent on the prescription of opioids postoperatively.

## Methods

A retrospective review was done on patients treated at a level 1 trauma center from January 2016 to December 2018. This study included patients younger than 18 years who had undergone one of the following nine surgical procedures unique to the pediatric population: closed reduction and percutaneous pinning or open reduction and percutaneous pinning of supracondylar humerus fracture (CPT: 24538, 24545-6), closed reduction and percutaneous pinning or open reduction and percutaneous pinning of humeral condylar or epicondylar fracture (CPT: 24582, 24579, 24575, 24566), flexible nailing of forearm fracture—radius and/or ulna (CPT: 25574 or 25575), slipped capital femoral epiphysis fixation (CPT: 27176, 27177, 27178, 27181), flexible nailing of femur fracture (CPT: 27506), closed reduction and spica cast application for femur fracture (CPT: 29305, 29325, 27502), open reduction and internal fixation of tibial tubercle (CPT: 27540 or 27535), flexible nailing of tibia fracture (CPT: 27758 or 27759), and open reduction and internal fixation of transitional distal tibia fracture (CPT: 27827 or 27828). Patients with polytrauma requiring more than one surgery during their hospitalization or those with more than one fractured extremity and lost to follow-up before the end of the global period were excluded. This study was approved by our center's institutional review board.

Patient data were obtained through billing query and chart review. Study variables included patient demographics (age, sex, race, and ethnicity), surgery done, length of stay, discharge prescriber type (advanced practice provider—nurse practitioner or physician assistant, resident, fellow, or attending physician), and discharge analgesic medications prescribed. For patients prescribed opioid medications (oxycodone is the only opioid medication used at our institution), the formulation (liquid or tablet), dosage, frequency, and number of doses prescribed were recorded. Morphine milligram equivalent prescribed was calculated for each patient. Patient phone calls and unplanned return visits to the emergency department or clinic for uncontrolled pain and opioid refills documented in our electronic medical record were also recorded. Refills were provided at the discretion of the treating provider. Our primary outcome variable was the number of opioid doses prescribed.

State-mandated preoperative opioid consenting for minors was signed into law on November 2, 2016, whereas consent publication online did not occur until February 4, 2017. Institutional uptake began by March 1, 2017, and broader adoption (>50% of cases) did not occur until June 1, 2017. Owing to this gradual implementation of the opioid consenting process, the presence of preoperative consent in the medical record was used as our predictor variable to represent law uptake in the presented univariate and multivariate analyses.

Because of the heterogeneity of ambulatory and inpatient procedures studied, evaluation of perioperative and inpatient pain management practices was not included as a part in this study. Anesthetic practices were not standardized and were at the discretion of the treating provider. Specifically, there is no routine utilization of regional anesthesia techniques in the management of orthopaedic trauma patients at our institution.

Descriptive statistics were used to analyze demographic information, procedures, prescribers, and prescribing practices. Bivariate testing, including *t*-tests, Mann-Whitney U tests, or Fisher exact tests, as appropriate, examined differences among patients with and without preoperative opioid consent. Assuming α = 0.05, β = 0.2, and SD of 21 doses, post hoc power analysis identified that a sample size of 192 patients in each consenting group was sufficient to detect a change in prescribing more than six doses of opioid medication (equaling approximately 1 day of medication based on q4 hour dosing). Univariate and multivariate negative binomial models reported as incidence rate ratios and 95% confidence intervals were done to identify factors associated with a greater number of prescribed opioid doses. Only those variables achieving significance in univariate analysis and baseline demographic characteristics (i.e., age and sex) were entered into multivariate models. All data analyses were done using IBM SPSS version 23 Statistics for Windows, Armonk, NY: IBM. For all analyses, *P* ≤ 0.05 denotes statistical significance.

## Results

A total of 1,793 patients with the mean age of 8.1 ± 5.2 years met inclusion criteria. Patients were grouped according to the presence of preoperative opioid consent with 967 patients (53.9%) in the consent group and 826 patients (44.9%) in the no consent group. Cohort demographics demonstrated no differences regarding sex, age, race, ethnicity, or weight (Table 1). In addition, procedural distribution between the groups was similar. A greater proportion of discharge prescribing was done by APPs in the consent group.

**Table 1:** Demographics, Procedural Distribution, and Prescribing Data

Variable	Total (n = 1793)	No Consent (n = 826)	Consent (n = 967)	*P*
Age (yrs)	8.10 ± 5.24	7.94 ± 4.66	8.12 ± 5.69	0.636
Sex	1083 (60.4)	492 (59.6)	591 (61.1)	0.467
Male	710 (39.6)	334 (40.4)	376 (38.9)	
Female				
Weight (kg)	35.67 ± 24.34	35.57 ± 24.75	35.33 ± 24.15	0.816
Race				
White/Caucasian	1033 (57.6)	475 (57.5)	558 (57.7)	0.398
Black/African American	403 (22.4)	192 (23.2)	211 (21.8)	
Asian	63 (3.5)	29 (3.5)	34 (3.5)	
Indian	16 (0.9)	10 (1.2)	6 (0.6)	
Multiracial	36 (2.0)	15 (1.8)	21 (2.2)	
Other	238 (13.3)	105 (12.6)	133 (13.7)	
Not reported	4 (0.2)	0 (0.0)	4 (0.4)	
Ethnicity				
Hispanic or Latino	151 (8.4)	66 (8.0)	85 (8.8)	1.00
Not Hispanic or Latino	1637 (91.2)	758 (91.8)	879 (90.9)	
Not reported	5 (0.4)	2 (0.2)	3 (0.3)	
Procedure				
CRPP/ORPP of supracondylar humerus Fracture	699 (40.0)	316 (38.3)	383 (39.6)	0.886
CRPP/ORPP of humeral condylar/epicondylar fracture	295 (16.5)	140 (16.9)	155 (16.0)	
Flexible nailing of forearm	168 (9.4)	72 (8.7)	96 (9.9)	
SCFE pinning	122 (6.8)	54 (6.5)	68 (7.0)	
Flexible nailing of femur	84 (4.7)	43 (5.2)	41 (4.2)	
Femur closed reduction and spica cast application	180 (10.0)	79 (9.4)	101 (10.4)	
Tibial tubercle ORIF	68 (3.8)	32 (3.9)	36 (3.7)	
Flexible nailing of tibia	76 (4.2)	38 (4.6)	38 (3.9)	
ORIF of transitional distal tibia fracture	101 (5.6)	52 (6.3)	49 (5.1)	
Length of stay (d)	1.29 (0.90-1.83)	1.44 (0.92-1.77)	1.18 (0.88-1.77)	**<0.001**
Discharge prescriber				
Advanced practice provider (NP/PA)	1062 (59.2)	424 (51.4)	638 (64.5)	**<0.001**
Resident	417 (23.3)	234 (28.3)	183 (18.9)	
Fellow	166 (9.3)	110 (13.3)	56 (5.8)	
Attending physician	28 (1.6)	14 (1.7)	14 (1.4)	
NSAIDs prescribed?				
Yes	868 (48.4)	191 (23.1)	677 (70.0)	**<0.001**
No	925 (51.6)	635 (76.9)	290 (30.0)	
Acetaminophen prescribed?				
Yes	1568 (87.5)	708 (85.7)	860 (88.9)	0.0501
No	225 (12.5)	118 (14.3)	107 (11.1)	
Opioids prescribed?				
Yes	1673 (93.3)	782 (94.7%)	891 (92.1)	**0.0378**
No	120 (6.7)	44 (5.3%)	76 (7.9)	
No. of opioids doses prescribed				
Liquid oxycodone (total mL/dose mL)	1175 (70.2)	29.10 ± 21.5	19.59 ± 20.72	**<0.001**
Oxycodone tablets	498 (29.8)	35.37 ± 18.37	27.63 ± 12.80	**<0.001**
Opioid refill?				
Yes	24 (1.4)	12 (1.5)	12 (1.3)	0.871
No	1649 (98.6)	770 (98.4)	879 (98.7)	

CRPP = closed reduction and percutaneous pinning, NP = nurse practitioner, NSAID = nonsteroidal anti-inflammatory drug, ORPP = open reduction and percutaneous pinning, ORIF = open reduction and internal fixation, PA = physician assistant

Cohort demographics and opioid prescribing before and after preoperative opioid consenting were implemented. Categorical variables are reported as frequency (%). Continuous variables are reported at mean ± SD or median (interquartile range). Bolded values represent significance in the presented analyses (p<0.05).

**Figure 1:**
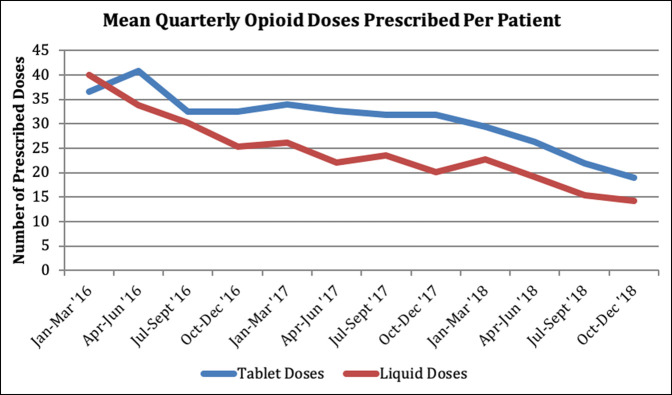
Graph showing mean quarterly opioid doses prescribed per patient in liquid and tablet formulation (2016 to 2018).

Overall, 70.1% of the patients received a liquid oxycodone prescription while the remaining 29.9% received tablet formulation. There was a significant decrease in the proportion of patients prescribed opioids in the consent group (94.7% versus 92.1%, *P* = 0.0378). This change was more apparent in the number of opioid doses dispensed, with a mean of 35.37 tablet and 29.10 liquid doses prescribed in the no consent cohort and 27.63 tablet (*P* < 0.001) and 19.59 liquid (*P* < 0.001) doses in the consent cohort. A downward trend was seen in the prescribing of both formulations over the study period (Figure 1). Additional subanalysis of the nine procedures studied demonstrated a notable decrease in both liquid and tablet doses prescribed in the consent group for most of the procedures. The consent group was also prescribed ibuprofen (*P* < 0.001) and acetaminophen (*P* = 0.05) more commonly and postoperative hospital length of stay was shorter (1.49 versus 1.77 days, *P* < 0.001). Postoperatively, we identified no change in the number of opioid refills requested within 30 days postoperatively between groups (Table 1).

In univariate analysis, significant predictors of fewer prescribed opioid doses were older age (*P* < 0.001), greater weight (*P* < 0.001), decreased length of stay (*P* = 0.020), prescription by an attending or fellow (*P* < 0.001 and *P* = 0.011), and preoperative informed consent for opioids (*P* < 0.001) (Table 2). An advanced practice provider prescriber (*P* < 0.001) was associated with more doses prescribed. In the multivariate analysis, age, weight, prescribing provider, and preoperative informed consent retained significance. Flexible nailing of forearm fractures was also associated with a lower volume of prescribed doses (*P* = 0.045).

**Table 2:** Predictors of Opioid Prescribing Practices

	Univariate Analysis		Multivariate Analysis	
Variable	IRR (95% CI)	*P*	IRR (95% CI)	*P*
Age	0.93 (0.91-0.94)	**<0.001**	0.96 (0.93 to 0.99)	**0.005**
Sex	0.88 (0.77-1.01)	0.071	Not entered	—
Weight (kg)	0.99 (0.98-0 0.99)	**<0.001**	0.99 (0.99-1.00)	**0.015**
Preoperative opioid consent obtained	0.89 (0.77-1.04)	**<0.001**	0.81 (0.68-0.96)	**0.004**
Acetaminophen prescribed postoperatively	1.12 (0.85-1.44)	0.442	Not entered	—
Ibuprofen prescribed postoperatively	1.00 (0.88-1.43)	0.944	Not entered	—
Length of stay (d)	1.07 (1.01-1.14)	**0.020**	—	NS
Discharge prescriber				
Advanced practice provider (NP/PA)	1.40 (1.17-1.66)	**<0.001**	1.77 (1.15 to 2.73)	**0.009**
Resident	0.93 (0.74-1.10)	0.321	Not entered	—
Fellow	0.45 (0.33-0.62)	**<0.001**	0.43 (0.26-0.69)	**<0.001**
Attending physician	0.42 (0.22-0.82)	**0.011**	0.15 (0.03-0.84)	**0.031**
Procedure				
CRPP/ORPP of supracondylar humerus fracture	1.42 (1.21-1.67)	**<0.001**	—	NS
CRPP/ORPP of humeral condylar/epicondylar fracture	1.05 (0.86-1.28)	0.725	Not entered	—
Flexible nailing of forearm fracture	0.67 (0.53-0.85)	**0.001**	0.73 (0.53-0.99)	**0.045**
SCFE pinning	0.66 (0.52-0.83)	**0.001**	—	NS
Flexible nailing of femur	1.60 (1.14-2.26)	**0.019**	—	NS
Femur closed reduction and spica casting	1.72 (1.26-2.34)	**0.001**	—	NS
Tibial tubercle ORIF	0.57 (0.40-0.81)	**0.002**	—	NS
Flexible nailing of tibia	1.21 (0.88-1.65)	0.241	Not entered	—
ORIF of transitional distal tibia fracture	0.57 (0.45-0.74)	**<0.001**	—	NS

CI = confidence interval, CRPP = closed reduction and percutaneous pinning, IRR = incidence risk ratio, NP = nurse practitioner, NS = not significant, NSAID = nonsteroidal anti-inflammatory drug, ORPP = open reduction and percutaneous pinning, ORIF = open reduction and internal fixation, PA = physician assistant

Negative binomial regression analysis of factors associated with the prescription of a greater number of postoperative doses of opioid medication. Bolded values represent significance in the presented analyses (p<0.05).

## Discussion

The recent opioid epidemic has placed increased emphasis on prescribing practices in the United States, particularly in the realm of orthopaedic trauma surgery.^1,6,7,9^ Fractures in the pediatric population are common and frequently require surgical treatment,^10,11^ and for many patients may represent a first exposure to opioid medications. These patients are subsequently at risk from the negative consequences of opioid-related adverse events as well potential misuse or medication diversion.^2-4,12,13^ In light of the growing concerns regarding early and prolonged opioid exposure in the pediatric population, this study was designed to assess trends in prescribing and evaluate associations between state-mandated preoperative opioid consenting and prescribing practices at a single center. We identified a notable decline in postoperative opioid prescribing over a 3-year period across nine common pediatric-specific orthopaedic surgical procedures, with preoperative consenting being a notable predictor of fewer doses prescribed across that time frame. Differences were greatest among liquid formulations. Patients undergoing consenting were also more commonly prescribed nonopioid medications, had shorter hospital length of stays while demonstrating no difference in need for opioid refills. These findings lend support to the use of preoperative opioid consent for minors as a means to affect prescribing practices without appreciable detriment to proxies of adequate postoperative pain control.

The finding of greatest clinical relevance in this study was the reduction in the number of prescribed opioid doses associated with the presence of preoperative opioid consent as demonstrated in our multivariate modeling. Patients undergoing medication consent received approximately 8 to 10 fewer doses of medication depending on formulation. This reduction in prescribing translates into nearly 9,000 fewer dispensed opioid doses or over 38,000 morphine milligram equivalents of medication (based on the mean prescribed liquid and tablet doses in this cohort), all of which would carry considerable risk of diversion^14^ and misuse in setting the frequent overprescription of these medications that have been found in pediatric surgery.^4,5^ These findings lend support for additional study and potential use opioid consenting as an intervention to improve opioid stewardship. Previous studies in other populations have investigated the implementation of other opioid-related mandates, such as prescription limits,^14-16^ and similarly found a decrease in the volume of prescribed opioids and the rate of opioid-related morbidity with these laws in place. Although the mechanism by which opioid consenting affects prescribing behaviors is uncertain, our theory is that the consenting process formalizes a discussion of the risks and benefits of these medications and forces providers and caregivers to critically evaluate their necessity for each patient and procedure. Although not directly addressed in this study, the consent process also educates caregivers and engages them in the decision-making process of medication prescribed. Their awareness of the risks of opioid use may further drive down consumption of these medications at home.

Another intriguing finding of our study beyond opioid prescribing practices was the parallel increase in written prescriptions for nonopioid analgesics, specifically ibuprofen and acetaminophen, after consent implementation. Both have long been used as part of multimodal therapy protocols and are a supplemental component of many established opioid prescribing guidelines.^17-19^ However, our findings suggest that a large proportion of providers in the early study period may have been relying on verbal or written instruction for these medications because they are available over-the-counter. Patients and caregivers, as a result, may be less likely to adhere to these instructions as closely as they would for a prescribed medication. After the implementation of opioid consenting, we identified a considerable shift toward the formal prescription of both of these nonopioid therapies. We feel that these changes may have contributed to the success of a simultaneous reduction in opioid prescribing and shorter hospital length of stay without a rise in opioid refill needs.

Variability in opioid prescribing in pediatric patients has been previously documented in the literature across a variety of other settings and surgical cohorts.^20-22^ The results from our study identified similar variability in prescribing practices across individual procedures and between prescribing provider types. Both of these findings support the need for didactic and standardization efforts to reduce variations that seem to lean toward overprescribing. Although some opioid guidelines have been proposed in the literature, most are limited to the management of chronic pain, adults, and/or nonorthopaedic procedures.^19,23-25^ Data related to opioid prescribing in pediatric orthopaedic trauma are limited to small patient cohorts, less procedural diversity, and study periods predating the declaration of an opioid public health emergency.^26,27^ This has left existing guidelines to make decisions regarding orthopaedic procedures solely based on “expert” opinion.^19,28^ Our findings provide a baseline prescribing trend across a large patient cohort and suggest a theoretical “upper limit” from which to start the development of prescribing guidelines. Continued efforts focusing on opioid stewardship at our own institution have further decreased prescription dosing below what is presented in this study. We believe that additional improvements in perioperative pain management practices can certainly be made to further drive down opioid prescribing needs and subsequent patient exposure.

This study is not without limitations, many of which are related to the retrospective design. First, while review of the medical record enabled identification of the type and volume of medications prescribed, it did not permit accurate tracking of opioid consumption. In addition, we were not able to directly assess the adequacy of pain control in the follow-up in a consistent manner across all procedures from chart review because many were ambulatory or required only a short inpatient stay and subsequently had to use opioid refill and unscheduled visits as a proxy. Future work using prospective, survey-based data capture^5^ would help to examine the actual consumption of opioids and pain control in real-time during the postoperative period. Next, it is important to note that the study period coincided with the emergence of the national “opioid crisis,” resulting in increased attention to opioid prescribing in the peer-reviewed literature and lay media. The growing awareness of the harms of opioid use likely placed increased pressure on healthcare providers to limit opioid prescriptions, which may have, in part, contributed in part to the gradual decline in prescribing over the study period. The timing of these effects, however, is nearly impossible to determine making their effect difficult to quantify. Finally, despite the large sample size and the diversity of procedures and prescribers included, the generalizability of the findings may be limited by the single-center design. Injuries with the same diagnosis and treatment occur on a spectrum, and as a level 1 trauma center and tertiary referral center, the conditions and subsequent pain control needs of this cohort may not parallel centers with lower acuity treating less severe patterns of these injuries.

Findings from this study highlight meaningful trends and define recent patterns of use in postoperative opioid prescribing across a large number of uniquely pediatric orthopaedic trauma procedures. In addition, our results indicate that preoperative opioid consenting was associated with opioid prescribing needs for postoperative pain management in pediatric orthopaedic trauma surgery. We believe that this supports the additional study of opioid stewardship interventions, such as preoperative opioid consenting to aid in the reduced expectation for and reliance on opioids in the postoperative setting in children.
